# Reversible hypocalcaemic cardiomyopathy following post-thyroidectomy hypoparathyroidism: a case report

**DOI:** 10.1093/ehjcr/ytag099

**Published:** 2026-02-04

**Authors:** Toan Quang Dang, Sy Van Hoang, Chieu Van Ly, Thuc Tri Nguyen

**Affiliations:** Department of Cardiology, Cardiovascular Center, Cho Ray Hospital, No. 201B, Nguyen Chi Thanh Street, Cho Lon Ward, Ho Chi Minh City 700000, Vietnam; Department of Cardiology, Cardiovascular Center, Cho Ray Hospital, No. 201B, Nguyen Chi Thanh Street, Cho Lon Ward, Ho Chi Minh City 700000, Vietnam; Department of Internal Medicine, School of Medicine, University of Medicine and Pharmacy at Ho Chi Minh City, No. 217, Hong Bang Street, Cho Lon Ward, Ho Chi Minh City 700000, Vietnam; Department of Cardiology, Cardiovascular Center, Cho Ray Hospital, No. 201B, Nguyen Chi Thanh Street, Cho Lon Ward, Ho Chi Minh City 700000, Vietnam; Department of Cardiology, Cardiovascular Center, Cho Ray Hospital, No. 201B, Nguyen Chi Thanh Street, Cho Lon Ward, Ho Chi Minh City 700000, Vietnam; Ministry of Health of Vietnam, No. 138A, Giang Vo Street, Giang Vo Ward, Hanoi 100000, Vietnam

**Keywords:** Dilated cardiomyopathy, Hypocalcaemia, Heart failure, Hypoparathyroidism, Reversible, Case report

## Abstract

**Background:**

Hypocalcaemic cardiomyopathy is a rare but reversible cause of dilated cardiomyopathy (DCM) and heart failure with reduced ejection fraction, commonly associated with post-operative hypoparathyroidism. Misdiagnosis as other forms of cardiomyopathy can delay appropriate treatment and worsen outcomes.

**Case summary:**

A 36-year-old male presented with progressive dyspnoea, abdominal pain, and peripheral oedema. He had a history of invasive thyroid carcinoma treated with total thyroidectomy and radioactive iodine therapy, complicated by permanent hypoparathyroidism. Despite sustained alcohol abstinence, he was misdiagnosed with alcoholic cardiomyopathy and experienced recurrent hospitalizations for heart failure despite guideline-directed medical therapy (GDMT). Evaluation revealed severe hypocalcaemia, prolonged QTc (557 ms), and echocardiographic findings of DCM with a left ventricular ejection fraction (LVEF) of 28%. Hypocalcaemic cardiomyopathy secondary to post-thyroidectomy hypoparathyroidism was diagnosed. Calcium and calcitriol supplementation, combined with GDMT, led to significant improvement. At 10 months, the patient’s LVEF improved to 52%, QTc normalized to 397 ms, and symptoms resolved completely. He was transitioned to endocrinology for long-term management.

**Discussion:**

Hypocalcaemic cardiomyopathy should be suspected in unexplained DCM with a history of hypoparathyroidism. Timely calcium testing and correction, along with GDMT, can reverse cardiac dysfunction and improve outcomes.

Learning points

- Severe hypocalcaemia due to post-thyroidectomy hypoparathyroidism is a rare but reversible cause of dilated cardiomyopathy and heart failure.
- Hypocalcaemic cardiomyopathy typically manifests as dilated cardiomyopathy and long QTc syndrome.
- Early calcium normalization and guideline-directed medical therapy can lead to significant reverse remodelling of cardiac structure and function, resulting in a favourable prognosis.

## Introduction

Hypocalcaemic cardiomyopathy is a rare and often overlooked cause of heart failure with reduced ejection fraction (HFrEF), typically associated with severe but reversible myocardial dysfunction when the underlying hypocalcaemia is appropriately corrected.^1^ This case explores hypocalcaemic cardiomyopathy resulting from untreated hypoparathyroidism, emphasizing the diagnostic challenges and successful therapeutic approach.

## Summary figure

**Figure:**
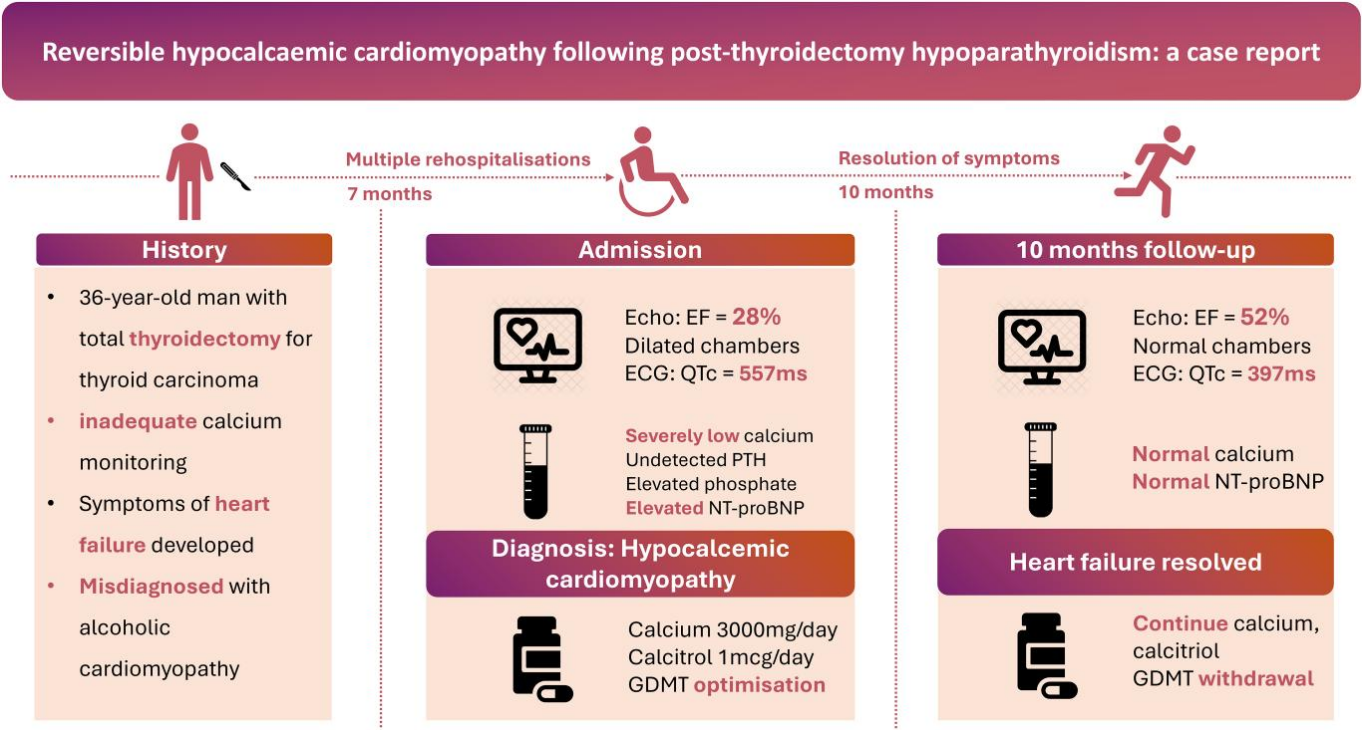


## Case presentation

A 36-year-old male presented to the emergency department with a 5-day history of abdominal pain, vomiting, diarrhoea, and progressive dyspnoea. His medical history was remarkable for invasive thyroid carcinoma treated with total thyroidectomy and central neck lymph node dissection 7 months earlier. Notably, preoperative echocardiography had shown a normal left ventricular ejection fraction (LVEF) of 68%. Post-operatively, he was prescribed levothyroxine (150 mcg/day), calcium carbonate (1000 mg/day), calcitriol (0.25 mcg/day), and vitamin D3 (250 IU/day).

Four months prior to admission, the patient developed progressive fatigue, dyspnoea, bilateral leg oedema, and weakness. Two months before presentation, he was diagnosed with HFrEF and dilated cardiomyopathy (DCM) attributed to alcoholic cardiomyopathy (ACM), due to a history of heavy alcohol consumption (300 g of ethanol daily since age 25) but had abstained from alcohol for 7 months following his thyroid cancer diagnosis. Despite initiation of guideline-directed medical therapy (GDMT), he experienced multiple rehospitalizations for worsening heart failure. There was no family history of cardiac or endocrine diseases.

On examination, the patient appeared fatigued but stable, with a blood pressure of 112/70 mmHg, heart rate of 78 b.p.m., temperature of 37.3°C, and oxygen saturation of 97% on room air. Cardiac auscultation revealed a grade 3/6 systolic murmur at the apex and bibasilar crackles were noted. Neurological examination revealed mild weakness (4/5) in all extremities, without Chvostek’s or Trousseau’s signs.

**Figure 1:**
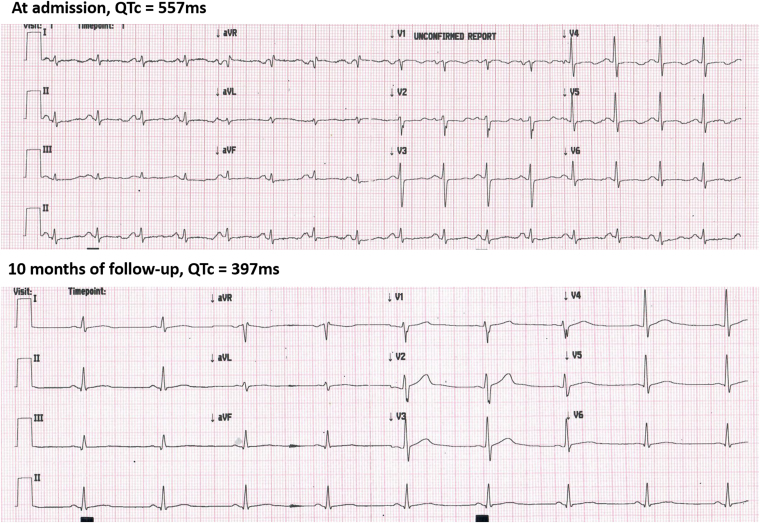
Top panel: Electrocardiography recorded at admission showing prolonged corrected QT interval (QTc) of 557 ms. Bottom panel: Electrocardiography recorded after 10 months of follow-up showing a normalized QT interval with a QTc of 397 ms following correction of the underlying hypocalcaemia.

**Figure 2:**
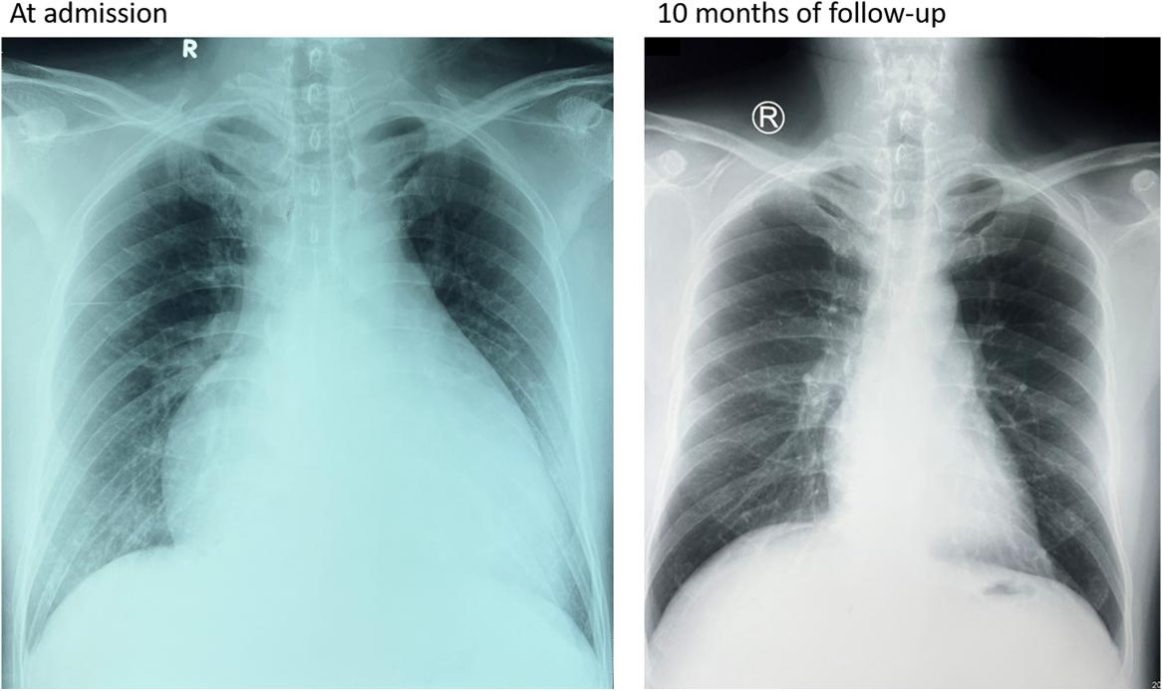
Left panel: Chest X-ray at admission showing cardiomegaly with an enlarged cardiac silhouette, indicative of severe left ventricular dysfunction. Right panel: Chest X-ray after 10 months of follow-up demonstrating normalization of the cardiac silhouette.

**Figure 3:**
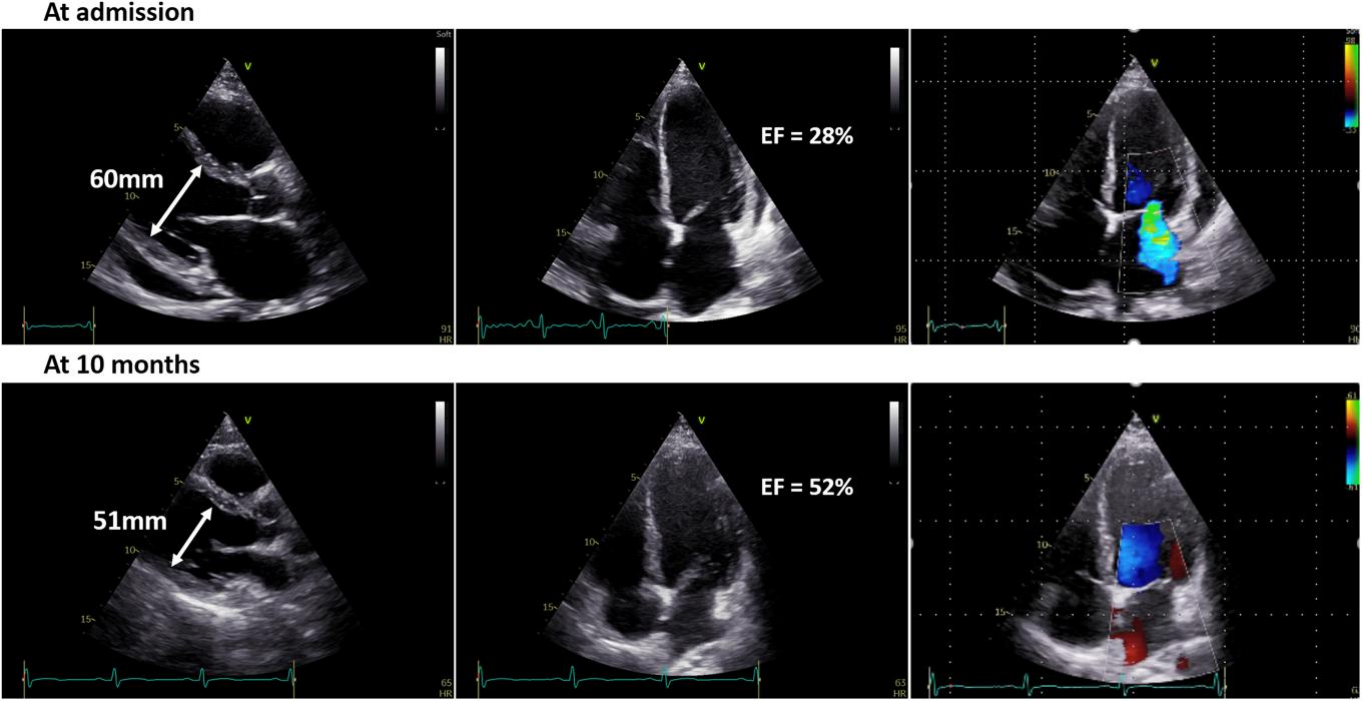
Top panel: At admission, left ventricular end-diastolic diameter was 60 mm, ejection fraction was 28%, and there were mitral regurgitation and pericardial effusion. Bottom panel: At 10 months, left ventricular end-diastolic diameter decreased to 51 mm, and ejection fraction improved to 52%, with resolution of mitral regurgitation and pericardial effusion.

**Figure 4:**
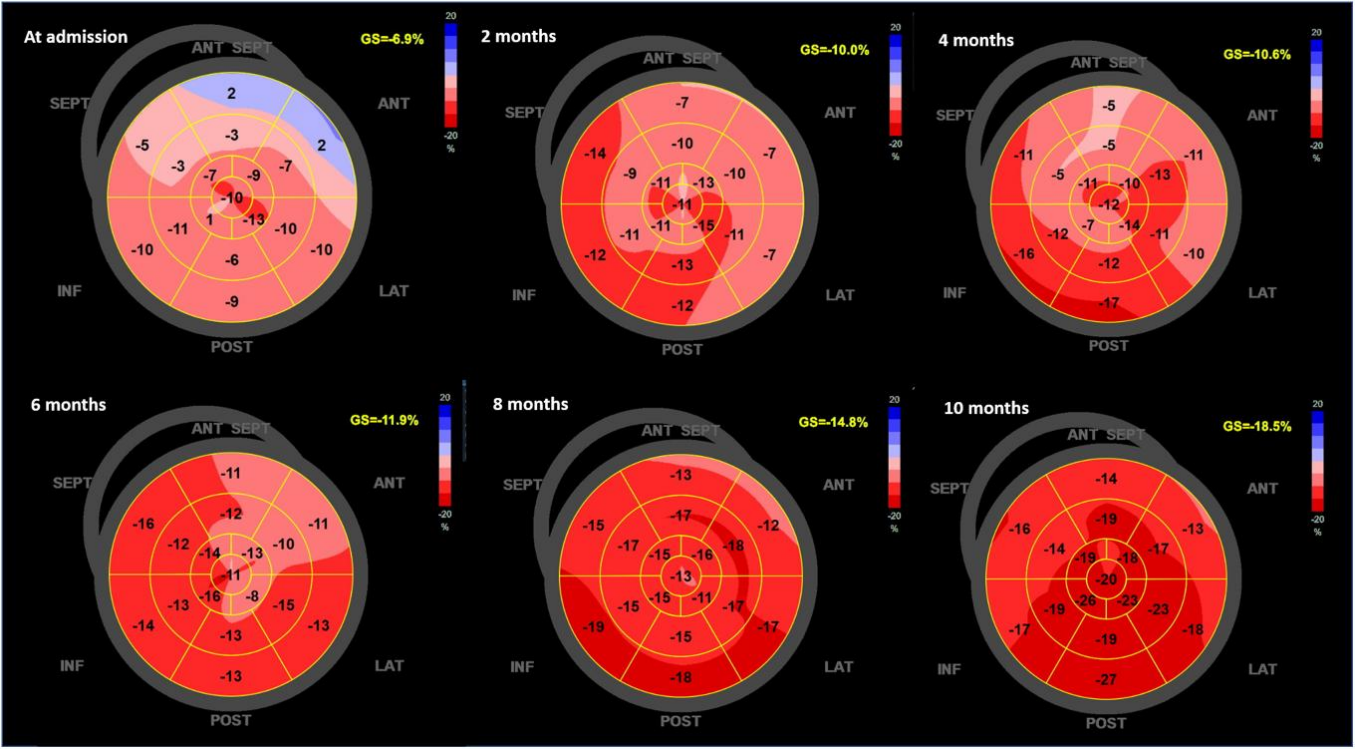
Global longitudinal strain analyses at admission and during follow-ups. The bull-eye plots demonstrate progressive improvement in global longitudinal strain over a 10-month period.

Initial investigations revealed severe cardiac dysfunction. Electrocardiography showed sinus rhythm with prolonged corrected QT interval (QTc) of 557 ms (*Figure 1*). Chest X-ray demonstrated cardiomegaly and interstitial oedema (*Figure 2*). Transthoracic echocardiography revealed dilated four chambers, severely reduced LVEF (28%), secondary mitral regurgitation, and pericardial effusion (*Figure 3*, *Table 1*, and *Video 1*). Speckle-tracking echocardiography showed severely reduced strain parameters, including a global longitudinal strain (GLS) of −6.9% (*Figure 4* and *Table 1*). N-terminal pro-B-type natriuretic peptide (NT-proBNP) was elevated at 1774.65 pmol/L, and further evaluation to determine the aetiology of DCM revealed severe hypocalcaemia (total calcium 0.9 mmol/L), undetectable parathyroid hormone (<4.6 pg/mL), and hyperphosphatemia (71.8 mg/L). Thyroid function tests were normal, and coronary angiography ruled out ischaemic heart disease (*Table 2*). These findings confirmed HFrEF due to hypocalcaemic cardiomyopathy caused by permanent hypoparathyroidism post-thyroidectomy.

**Table 1:** Echocardiography results

Parameters	At admission	At 10 months
LVEF (%)	28	52
LVEDd (mm)	60	51
LVEDi (mL/m^2^)	76.6	50
LAVI (mL/m^2^)	42.05	19.48
RAVI (mL/m^2^)	54.35	22.05
RV longitudinal length (mm)	91	75
GLS (%)	−6.9	−18.5
LArs (%)	7	32
RArs (%)	12	27
RVFWS (%)	−7	−21
Mitral regurgitation	Moderate to severe	None
Pericardial effusion	Moderate	None

LVEF, left ventricular ejection fraction; LVEDd, left ventricular end diastolic diameter; LVEDi, left ventricular end diastolic index; LAVI, left atrial volume index; RAVI, right atrial volume index; RV, right ventricular; GLS, global longitudinal strain, LArs, left atrial reservoir strain, RArs, right atrial reservoir strain, RVFWS, right ventricular free wall strain.

**Table 2:** Laboratory data

Test	At admission	At 10 months	Normal range
Total calcium (mmol/L)	**0**.**9**	2.3	2.2–2.6
Ionized calcium (mmol/L)	**0**.**42**	1.18	1–1.5
Parathyroid hormone (pg/mL)	**< 4.6**		18.5–88
Phosphate (mg/L)	**71**.**8**		25–42
25-Hydroxyvitamin D (nmol/L)	74.6		25–125
N-terminal pro-B-type natriuretic peptide (pmol/L)	**1774**.**65**	13.16	<14.5
High-sensitivity troponin I (pg/mL)	**63**.**6**		<34.2
Free thyroxine (pg/mL)	20.89	13.3	8–20
Free triiodothyronine (pg/mL)	1.87	1.93	1.5–4.2
Thyroid-stimulating hormone (mIU/L)	9.73	3.87	0.4–5
C-reactive protein (mg/L)	**7**		<6
Urea (mg/dL)	14	18	7–20
Creatinine (mg/dL)	1.32	1.05	0.7–1.5
Estimated glomerular filtration rate (mL/min/1.73 m^2^)	**68**.**91**	90.88	≥90
Sodium (mmol/L)	138	140	135–150
Potassium (mmol/L)	3.5	3.5	3.5–5.5
Chloride (mmol/L)	93	99	98–106
Magnesium (mmol/L)	0.84	0.83	0.7–1.2
Aspartate transaminase (U/L)	38		9–48
Alanine transaminase (U/L)	21		5–49
Albumin (g/dL)	4.8		3.5–5.5
Protein (g/dL)	7.1		6–8

Bold values indicate abnormal results.

The patient received immediate treatment with intravenous calcium chloride (1000 mg), followed by oral calcium gluconate, calcitriol, and GDMT. His symptoms improved rapidly, and he was discharged on Day 6. During follow-ups, oral calcium gluconate (3000 mg/day) and calcitriol (1 mcg/day) were titrated to maintain normal calcium levels, and GDMT was optimized, including sacubitril/valsartan 200 mg b.i.d., metoprolol succinate 150 mg o.d., spironolactone 50 mg o.d., and empagliflozin 10 mg o.d.

At a 10-month follow-up, the patient showed markedly improvement. Left ventricular ejection fraction increased to 52%, GLS improved to −18.5%, and chamber dimensions normalized (*Figures 3* and *4*, *Table 1*, and *Video 2*), NT-proBNP decreased to 13.16 pmol/L (*Table 2*), QTc normalized to 397 ms (*Figure 1*), and chest X-ray showed normal cardiac silhouette (*Figure 2*). Calcium and calcitriol supplementation were continued, while GDMT was gradually discontinued. The patient resumed normal activities, and he was referred to endocrinology for long-term management of hypoparathyroidism.

## Discussion

We report a 36-year-old male with recurrent hospitalizations for heart failure, initially misdiagnosed as ACM despite sustained abstinence. He presented with progressive heart failure symptoms, prolonged QTc, and severely reduced LVEF. Investigations revealed severe hypocalcaemia and undetectable parathyroid hormone, confirming hypocalcaemia-induced cardiomyopathy secondary to permanent hypoparathyroidism post-thyroidectomy. Treatment with calcium, calcitriol, and GDMT resulted in rapid improvement and full recovery at 10 months.

Hypocalcaemia-induced DCM is a rare but important cause of heart failure. The primary causes of hypocalcaemia include hypoparathyroidism, vitamin D deficiency, chronic kidney disease, and malabsorption syndromes.^1,2^ Clinical manifestations typically include heart failure symptoms, prolonged QTc, and HFrEF, mimicking idiopathic DCM.^1^ This underscores the importance of considering hypocalcaemia in patients with unexplained heart failure, particularly those with risk factors for calcium dysregulation.

Calcium plays a critical role in myocardial contractility and electrophysiology, and hypocalcaemia impairs excitation–contraction coupling,^3^ prolongs QTc,^4^ and promotes ventricular remodelling.^5^ Hence, early recognition and correction are essential to prevent irreversible myocardial damage.

Diagnosis is straightforward with serum calcium testing, but clinical suspicion is crucial in high-risk scenarios such as post-thyroidectomy. According to the ESC 2023 guidelines, calcium testing is recommended as a first-line investigation in patients with DCM to identify reversible causes.^6^ Key findings include low serum calcium, prolonged QTc, and left ventricular dilation and dysfunction. Exclusion of other DCM causes, such as ischaemic heart disease, is essential.^1^ In this case, lack of regular calcium monitoring post-thyroidectomy led to severe hypocalcaemia, delayed diagnosis, and misdiagnosis as ACM.

The prognosis of hypocalcaemia-induced DCM is highly favourable with timely treatment. Calcium supplementation restores myocardial function in 94% of cases, with nearly 75% achieving complete normalization of ejection fraction.^2^ However, delayed diagnosis or prolonged hypocalcaemia may result in irreversible myocardial damage, emphasizing the importance of early intervention.^7^

Treatment involves calcium and calcitriol supplementation to restore normocalcaemia and improve myocardial function.^8^ In acute settings, intravenous calcium is preferred to rapidly correct severe hypocalcaemia, particularly in patients presenting with cardiogenic shock. Once stabilized, oral calcium supplementation combined with calcitriol is used to maintain calcium levels within the normal range. Notably, loop diuretics, mostly used in acute phase, can exacerbate hypocalcaemia, while thiazide is safer in this context.^8^

Calcium supplementation alone can lead to partial or complete recovery of cardiac function, depending on the duration of hypocalcaemia and the extent of myocardial damage.^2^ However, prolonged exposure to hypocalcaemia may result in irreversible damage due to persistent myocardial stress and structural changes.^7^ Guideline-directed medical therapy plays a critical role in promoting reverse remodelling, even in cases with reversible causes.^9^ In this case, the use of GDMT likely contributed to the patient’s recovery and should be considered in similar cases to support cardiac remodelling.

Long-term follow-up is essential to ensure sustained recovery and prevent recurrence. Continued calcium and calcitriol supplementation is mandatory in permanent hypoparathyroidism to maintain normocalcaemia and prevent further episodes of heart failure. While the TRED-HF trial demonstrated unfavourable outcomes with GDMT withdrawal in recovered DCM, cases of DCM with a known and treatable cause, such as hypocalcaemia, may allow for GDMT discontinuation once cardiac function normalizes.^10^ In this case, the successful discontinuation of GDMT supports this approach, but further research is needed to establish its safety in similar scenarios.

In conclusion, this case highlights the importance of recognizing hypocalcaemia as a rare but treatable cause of DCM. Routine calcium testing in unexplained DCM, especially post-thyroidectomy, enables early diagnosis. Timely calcium and calcitriol supplementation can significantly restore cardiac function, while GDMT supports cardiac remodelling and improved outcomes, though further research is needed on the safety of its withdrawal in treatable DCM cases.

## Lead author biography


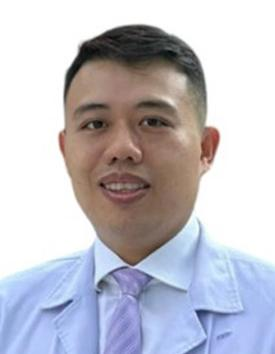


Dr Toan Quang Dang is a dedicated cardiologist at Cho Ray Hospital, specializing in heart failure, cardiomyopathies, and echocardiography. He is committed to providing high-quality care for patients with complex heart conditions.

## Data Availability

Data availability is not applicable, as this case report did not generate or analyse any new datasets.
